# Optical and Electrochemical Applications of Li-Doped NiO Nanostructures Synthesized via Facile Microwave Technique

**DOI:** 10.3390/ma13132961

**Published:** 2020-07-02

**Authors:** Aarti S. Bhatt, R. Ranjitha, M. S. Santosh, C. R. Ravikumar, S. C. Prashantha, Rapela R. Maphanga, Guilherme F. B. Lenz e Silva

**Affiliations:** 1 Department of Chemistry, N.M.A.M. Institute of Technology (Visvesvaraya Technological University, Belagavi), Nitte 574110, India; aartis@nitte.edu.in; 2 Department of Chemistry, St. Aloysius College (Autonomous), Mangaluru 575003, India; ranjut85@gmail.com; 3 Centre for Incubation, Innovation, Research and Consultancy (CIIRC), Jyothy Institute of Technology, Thataguni, Off Kanakpura Road, Bangalore 560082, Karnataka, India; 4 Research Centre, Department of Chemistry, East West Institute of Technology, Bengaluru 560091, India; scphysics@gmail.com; 5 Next Generation Enterprises and Institutions, Council for Scientific and Industrial Research, Pretoria 0001, South Africa; RMaphanga@csir.co.za; 6 Department of Physics, University of Limpopo, Private bag x 1106, Sovenga 0727, South Africa; 7 Polytechnic School of Engineering, University of São Paulo, São Paulo 05508-030, Brazil; guilhermelenz@usp.br

**Keywords:** Li-doped NiO, microwave synthesis, computational simulation, electrochemical measurements, photoluminescence

## Abstract

Nanostructured NiO and Li-ion doped NiO have been synthesized via a facile microwave technique and simulated using the first principle method. The effects of microwaves on the morphology of the nanostructures have been studied by Field Emission Spectroscopy. X-ray diffraction studies confirm the nanosize of the particles and favoured orientations along the (111), (200) and (220) planes revealing the cubic structure. The optical band gap decreases from 3.3 eV (pure NiO) to 3.17 eV (NiO doped with 1% Li). Further, computational simulations have been performed to understand the optical behaviour of the synthesized nanoparticles. The optical properties of the doped materials exhibit violet, blue and green emissions, as evaluated using photoluminescence (PL) spectroscopy. In the presence of Li-ions, NiO nanoparticles exhibit enhanced electrical capacities and better cyclability. Cyclic voltammetry (CV) and electrochemical impedance spectroscopy (EIS) results show that with 1% Li as dopant, there is a marked improvement in the reversibility and the conductance value of NiO. The results are encouraging as the synthesized nanoparticles stand a better chance of being used as an active material for electrochromic, electro-optic and supercapacitor applications.

## 1. Introduction

Nano metal oxides are attracting several researchers due to their varied applications. The reduction in their size contributes tremendously to expanding the surface area, thereby making their optical and electrical properties highly sensitive to surface morphology. Among the various nanometal oxides, nickel oxide (NiO) nanoparticles have been extensively investigated because of their excellent chemical stability and favourable opto-electrical properties. NiO is an antiferromagnetic material and possesses a cubic structure. It has a band gap in the range of 3.6–3.8 eV [1] which can be tailored by reducing the size and/or by doping. It is therefore not surprising that NiO finds applications as antiferromagnetic materials [2], p-type transparent conducting films [3], electrochromic display materials [4] and chemical sensors [5].

For electro-optic applications, the stoichiometry of the crystal and optimization of the band structure are crucial. The chemical composition and crystal structure of the nanoparticles can be manipulated by introducing impurity atoms. The presence of dopants alters the energy configuration of the crystal lattice, thereby influencing the optical and transport properties. Generally, dopants from I to V group elements are introduced to obtain a stable p-type NiO semiconductor [6]. It has been observed that the presence of Li in the metal oxide lattice increases the electrical resistivity, thereby making them suitable for manufacturing transparent conducting oxides, piezoelectric devices and memory devices [7,8]. However, it is also claimed [9,10,11] that the presence of Li as a dopant results in a decrease in the resistivity of NiO leading to an improvement in its electrochromic properties. Hence, it becomes necessary to understand the effect of dopant concentration on the electrochemical properties.

The optical properties of the materials also play a crucial role in several applications, like optoelectronics, integrated optics, solar power engineering and optical sensor technology. Generally, compounds like C_2_S, Ca_2_SiO_4_ and CaAl_2_O_4_ are considered as standard phosphor materials [12,13,14,15]. It is reported that doping of P ions in the green emitting C_2_S:Eu^2+^ effectively enhances the PL intensities [13]. Doping these with rare earth metals like Eu and Nd is known to enhance their emission characteristics. Nakano et al. have reported the effect of annealing temperature on the photoluminescence of Eu doped Ca_2_SiO_4_. They observed a red emission at 1773 K and green emission at 1473 K [14]. Recently, a significant work on Eu/Nd-doped Ca_12_Al_14_O_33_ and CaAl_2_O_4_ phosphors as long-lasting blue emitters has also been carried out [15]. Though binary metal oxides are well known for their superior luminous efficiency, related works on simple oxides are rare. Wide gap semiconductors like ZnO have been used extensively as nanophosphors [16,17]. It was found that the doping of these nanophosphors with dopants like Li [18], Al [19], Mg [20], V [21], Eu [22] and Er [23] significantly alters their visible emission spectra. It is interesting to note that the number of research papers reporting on the electrochemical properties of NiO materials are much greater in comparison to the reports on their optical properties. NiO, being a p-type semi-conductor, stands a fair chance of being employed as a light-emitting diode or a photodetector in photoelectronic devices. To the best of our knowledge, there are very few studies [18] exploring the effect of Li ion on the optical properties of NiO. The present work is an attempt to underline the effect of Li in tuning the optical behavior of NiO along with its electrochemical characteristics.

In this background, the present work is focused on understanding the influence of Li doping on the structural, optical and electrochemical properties of NiO. The nanoparticles were synthesized by microwave-assisted synthesis. Since here, high-frequency microwaves are used for heating, this method has an advantage over conventional heating of being faster and consuming low energy. Although there is literature available on the microwave synthesis of NiO [24,25,26] and doped NiO [27], to the best of our knowledge, the present work is the first attempt to synthesize Li-doped NiO by microwave radiations. A detailed mechanism has also been proposed to understand the decrease in the band gap and resistivity of the as-synthesized nanomaterials on inclusion of the dopant.

## 2. Materials and Methods

### 2.1. Synthesis of NiO Nanoparticles

Nickel acetate tetrahydrate, lithium carbonate, polyethylene glycol 200 (PEG 200) and sodium hydroxide pellets were obtained from Sigma Aldrich, Bangalore, India. All chemicals used in this work are of analytical grade and were used without any further purification. An aqueous solution of 0.2 M nickel acetate was prepared. To this, 0.3 M NaOH solution was added dropwise with continuous stirring. The mixture was stirred for an hour to achieve concentration homogeneity. The resulting green-colored solution was heated in a microwave oven for 5 min at 210 W and 95 °C. The green-colored product thus obtained was alternately washed with water and ethanol till a pH of 7.0 was attained. The precipitate was separated by centrifugation and the solid precursor obtained was annealed in a muffle furnace at 300 °C for 2 h.

**Figure 1:**
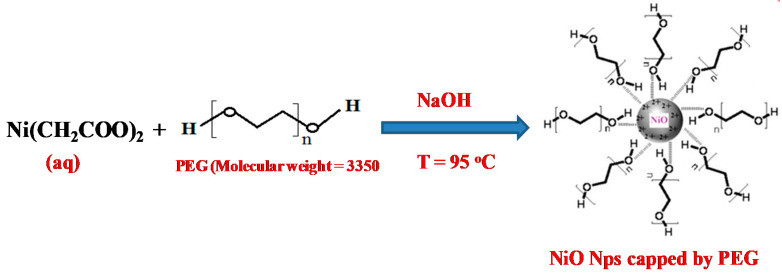
Structural impact of nickel acetate and polyethylene glycol(PEG) reaction.

**Figure 2:**
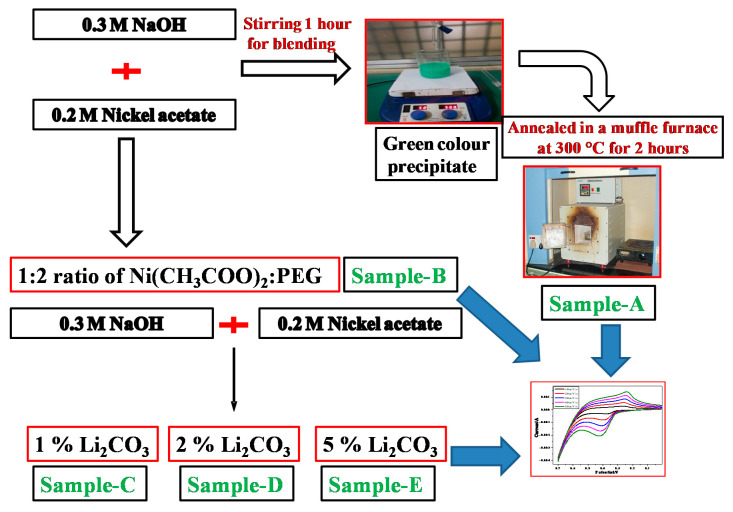
Flow chart for the synthesis of different NiO nanoparticles.

In an alternate procedure, PEG 200 which acts as a surfactant, was dissolved in NaOH solution before adding it dropwise to nickel solution in a similar manner described earlier. The surfactant to Ni(CH_3_COO)_2_ ratio was fixed to 2:1. It can be inferred that the surfactant acts on the surface of NiO nuclei, thereby inhibiting its growth (Figure 1). In a similar manner, doped samples were prepared by adding lithium carbonate solutions of different concentrations (1, 2 and 5%) to nickel acetate solutions, followed by alkali addition. The overall idea of the synthetic method adapted is depicted in the flow chart represented in Figure 2.

### 2.2. Characterization

X-ray diffraction studies were carried out on Philips X’pert PRO X-ray diffractometer, Malvern, UK, with graphite monochromatized CuK_α_ (λ = 1.5418 Å) radiation, at a scan rate of 10° min^−1^. Surface morphology was studied using a Field Emission Scanning Electron Microscopy (FESEM; Neon 40 Crossbeam, Carl Zeiss, Jena, Germany) with a resolution of 1.1 nm. Diffuse reflectance spectra were examined using the Shimadzu UV-VIS spectrophotometer (UV-2600, Kyoto City, Japan), in the range of 200–800 nm. Photoluminescence studies were carried out on a Horiba (model Fluorolog-3) spectrofluorimeter (Kyoto City, Japan) with 450 W Xenon excitation source. The spectral analysis was carried out using Fluor Essence™, Version 3.9.

### 2.3. Preparation of Carbon Paste Electrode for Electrochemical Studies

Cyclic voltammetry (CV) and electrochemical impedance spectroscopy (EIS) were performed on Model CHI604E potentiostat (CH Instruments, TX, USA). The experiment was carried out in acell comprised of a three-electrode system—a carbon paste working electrode (3.0 mm in diameter), a platinum wire counter electrode and a saturated Ag/AgCl reference electrode. The carbon paste electrode was prepared by grinding 70% graphite powder (particle size 50 µm and density 20 mg/100 mL), 15% of the prepared sample (A to E in Figure 2) and 15% silicone oil. The resulting homogeneous product was packed into the cavity of a customized polymer tube and smoothened at the ends.

### 2.4. Computational Details

The total energies were calculated by the projected augmented planewave (PAW) of density function-al theory, as implemented in the CASTEP code embedded in the Materials Studio software, Version 5.5.2 [28]. The core electrons were described using the projector augmented method. The exchange–correlation energy of the electrons was treated using the generalized gradient approximation within the Perdew–Burke–Ernzerhof functional [29]. For geometry optimization, the Monkhorst–Pack scheme was used. Ground state geometries were calculated by minimizing stresses and Hellman–Feynman forces using the conjugate gradient algorithm with the convergence parameters set as follows: total energy tolerance 2 × 10^−6^ eV/atom, maximum force tolerance 0.05 eV/nm and maximum stress component 0.1 GPa. From the convergence test calculations, the basis set kinetic energy cutoff of 600 eV was sufficient to ensure optimum accuracy in the computed results. The dimensions for nanoclusters were set 20.00 × 20.00 × 20.00 Å, large enough to ensure that there were no interactions between the system and its self-image along all the axes within the periodic boundary conditions. Different sizes of the nanoparticles were constructed from the optimized bulk NiO system using supercells of varying sizes. The NiO nanoparticle was constructed by cleaving the bulk system along the (110) plane. Afterward, the Li ions were inserted into the nanoparticles using two different doping mechanisms, i.e., interstitial and substitutional doping. The k-points were generated using the Monkhorst-Pack method with a grid size of 1 × 1 × 1 for structural optimization for nanoparticle. The stoichiometry was maintained throughout nanoparticle construction and the vacuum was included in the x, y and z directions. The vacuum thickness was considered wide enough to prevent nanoparticle-to-nanoparticle interactions and 20 Å was sufficient to ensure that the energy converged to less than 1 meV/atom.

## 3. Results and Discussion

### 3.1. XRD Analysis

**Figure 3:**
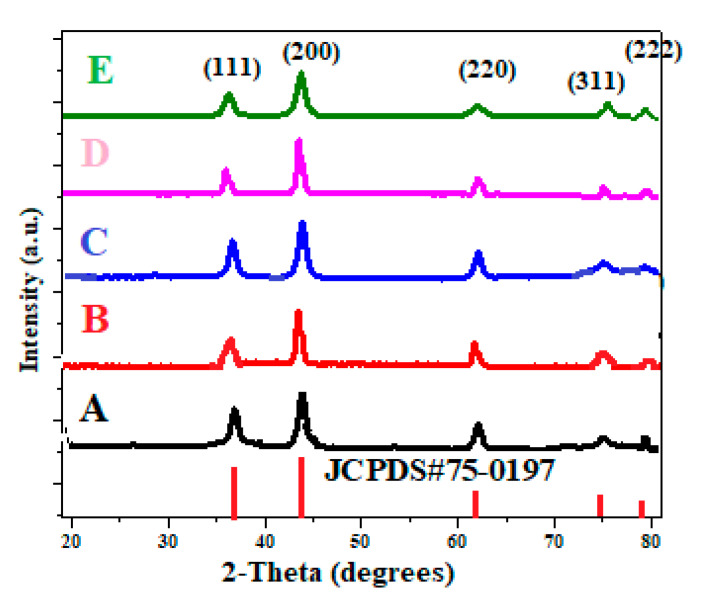
XRD pattern of A–E samples.

X-ray diffraction analysis was carried out to investigate the structures of pure NiO and Li doped NiO samples. Figure 3 shows the XRD peaks of pure NiO and samples prepared with (Sample B) and without (Sample A) surfactant, along with Li doped NiO (Samples C, D and E). The XRD patterns reveal the polycrystalline nature of the samples. The observed 2*θ* values are in good agreement with the standard JCPDS data (Card ID 75-0197). All the peaks are well indexed, with the favoured orientations being (111), (200) and (220) planes [23]. From the analysis of the peak positions and comparative intensities of the diffracted peaks, it was confirmed that the samples were monophasic, Fm-3m cubic NiO. Since the sample is in powder form, Scherrer equation (Equation (1)) was used to relate the size of the crystallites to the broadening of the peak. The crystallite sizes, evaluated using the (200) peak as reference, are tabulated in Table 1.

(1) τ=Kλβcosθ

where *τ* is the mean size, *K* is a dimensionless shape factor with a value of 0.94, *λ* is X-ray wavelength, *β* is the FWHM value and *θ* is the Bragg angle. From Table 1, it can be inferred that the presence of PEG during the synthesis of nanoparticles inhibits grain growth, as manifested by the peak broadening. PEG plays a crucial role in modifying the surface properties, thereby arresting grain growth. It is also observed that the Li doping of NiO also modified its crystallite size. This may be mainly due to the slightly larger size and lower charge of Li^+^ compared to that of the Ni^2+^; ionic radii of Li^+^ and Ni^2+^, which are 0.74 Å and 0.69 Å, respectively [30]. This suggests that, upon doping with Li, the dopant ions enter the rock salt crystal lattice of NiO substitutionally, and the surface stress may retard the growth of NiO nanomaterials. However, what is curious is that, at larger dopant concentrations (at 5%), this behaviour is not so pronounced. As the concentration of dopant increases, the peak intensity increases, revealing the higher crystallinity of the samples. This is reflected in the gradual increase in the crystallite size of the doped samples. Because of the low activation energy and high ionic mobility, the Li ions enter the nucleation sites, leading to increased strain and grain size [31]. A similar trend was observed by Matsubara et al. while doping NiO with Li up to 15 wt.%, who attributed this to the small amount of lattice contraction of NiO matrix on efficient substitution of Li^+^ for Ni^2+^ [32].

### 3.2. Surface Morphology

**Figure 4:**
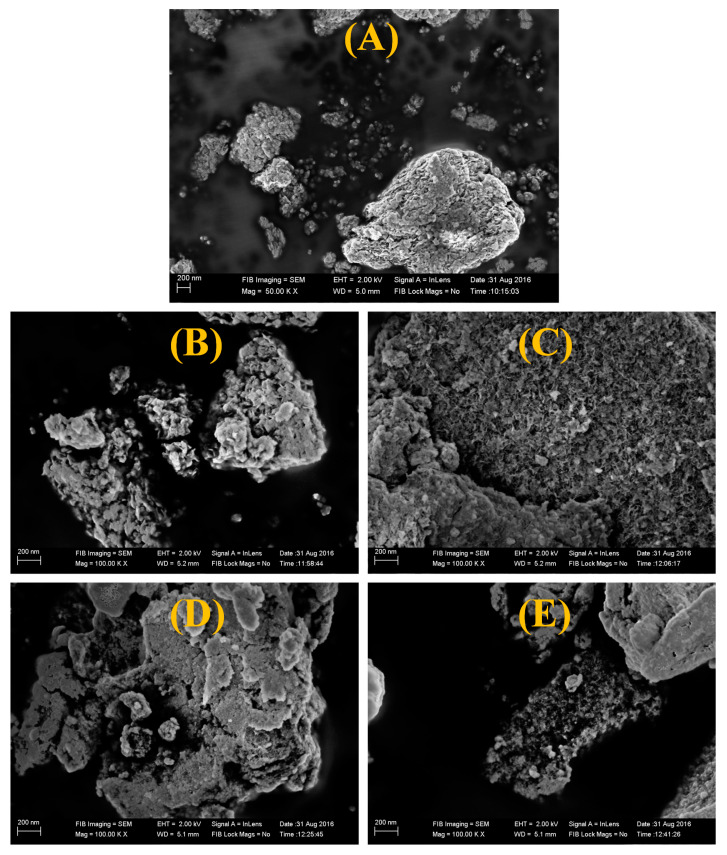
SEM images of (**A**) pristine NiO, (**B**) pristine NiO with PEG, (**C**) NiO with PEG and 1 wt% Li, (**D**) NiO with PEG and 2 wt% Li, (**E**) NiO with PEG and 5 wt% Li.

Figure 4 shows the FESEM images of pure NiO and Li-doped samples with different concentrations of Li. It is clear that the NiO particles are of spherical shape with a size distribution in the 50–100 nm range. It is evident that the obtained NiO particles are porous in nature. This suggests that since, during the synthesis, a large amount of gases evolve, this results in the disintegration of agglomerates and non-uniform dissipation of heat within the system. This can hinder the grain growth and eventually lead to an increase in the surface area and porosity of the material. When compared to the undoped samples, the doped samples exhibit a more uniform micrograph. For samples C, D and E, the small crystallite size leads to agglomeration. However, as the dopant concentration increases, the particles display a more prominent spherical shape.

### 3.3. Computational Studies

**Figure 5:**
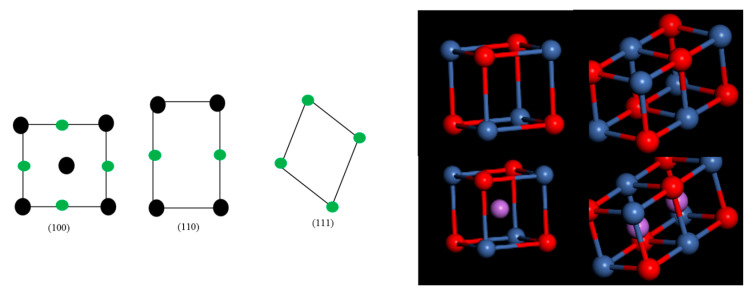
Constructed nanoparticles for NiO and Li-inserted NiO.

Geometrically, NiO is a stacked rocksalt structure with a space group Fm-3m and lattice parameter 4.17 Å [33]. Three possible surface orientations for NiO can be obtained, which are (100), (110) and (111). The (100) is the most stable surface, with the surface energy of 1.15 J/m^2^ [34]. The constructed nanoparticles for NiO and Li-inserted NiO are depicted in Figure 5 and have an octahedron geometry with a diameter of 3 nm. All nanoparticles possess a high symmetry, and their surfaces are characterized by (100) Miller index. A recent study investigating the effects of NiO nanoparticle surface energies on catalytic efficiency showed that (110) and (111) dominate the nanoparticle surface [35]. The Li doping of NiO has an impact on its crystal structure and also crystal size.

Computational modelling of optical functions for solids gives insights into understanding the optical properties of different materials. Due to the limitations and failure of GGA to capture the properties of strongly correlated electronic systems such as antiferromagnetic NiO, the simulated energy band gaps for the nanoparticles were significantly underestimated. The SCF energy is sensitive to the pseudopotential as well as the exchange and correlation functional used in the DFT calculation, hence the relative energies of simulated NiO nanoparticles are dependent on ground state energy.

The frequency-dependent dielectric function is given by:

(2) $$\varepsilon \;\left(\omega \right)=\varepsilon_{1}\left(\omega \right)+i\varepsilon_{2}(\omega )$$

The dielectric function is closely related to the electronic band structure and it fully describes the optical properties of any homogeneous medium at all photon energies. The imaginary part of the complex dielectric function is obtained from the momentum matrix elements between the occupied and unoccupied electronic states. The imaginary part is calculated using the analytical expression

(3) $$\varepsilon_{2}\left(\omega \right)=\frac{2e^{2}\pi }{\Omega \varepsilon_{0}}{\sum_{k,v,c}\left|\langle \psi_{k}^{c}|\hat{u}\cdot \vec{r}|\langle \psi_{k}^{v}|\right|}^{2}\delta \left(E_{k}^{c}-E_{k}^{v}-E\right)$$

where ω is the frequency of light, *e* is the electronic charge, $\hat{u}$ is the vector defining the polarization of the incident electric field, $\psi_{k}^{c}$ and $\psi_{k}^{v}$ are the conduction and valence band wave functions at *k*, respectively. The real part of the dielectric function $\varepsilon_{1\;}\left(\omega \right)$ is derived from the imaginary part of the dielectric function $\varepsilon_{2}\;\left(\omega \right)$ through the Kramers–Kronig relationship. Other optical properties, such as refractive index, absorption spectrum, loss function, reflectivity, and conductivity (real part), are derived from

(4) $$\varepsilon_{1}(\omega )=1+\frac{2}{\pi }P\int_{0}^{\infty }\frac{\omega^{\prime }\varepsilon_{1}(\omega^{\prime })d\omega^{\prime }}{\left({\omega^{\prime }}^{2}-\omega^{2}\right)}$$

### 3.4. Diffuse Reflectance Spectral (DRS) Analysis

**Figure 6:**
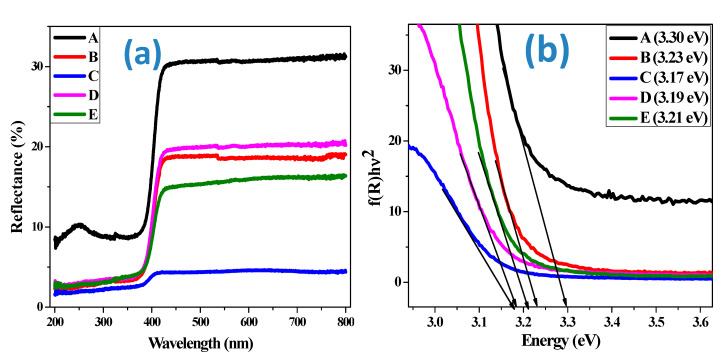
(**a**) Diffuse reflectance spectra of NiO samples. (**b**) Optical energy gap spectra of NiO samples.

The reflectance, transmittance and scattering of the synthesized materials were determined using diffuse reflectance spectra. Figure 6 illustrates the diffuse reflectance spectra of different NiO samples. It can be seen from Figure 6a that each sample exhibits an absorption edge and the reflectance decreases with the addition of Li. The spectra were further used to plot the optical energy gap spectra of NiO samples as indicated in Figure 6b. The band gap decreases from 3.3 eV (undoped NiO) to a minimum of 3.17 eV (NiO doped with 1% Li). The variation in the band gap cannot be explained plainly on the basis of Quantum Size Effect. This is because, as seen from the XRD data, the particle size obtained is beyond quantum size confinement.

With an octahedral symmetry, the divalent nickel ions possess a 3d^8^ electronic configurations and the spin-allowed d–d transitions of octahedral Ni^2+^ ions are because of the single broad absorption band visible at 410 nm. As the powder sample diffuses the light in large quantities with a greater thickness, the absorption spectra becomes more complicated to interpret. In order to minimize this difficulty, DRS along with Schuster–Kubelka–Munk (SKM) relation has been used [36,37] which can be given as

(5) $$F\left(R\right)=\frac{{\left(1-R\right)}^{2}}{2R}$$

where *R* is the absolute reflectance of the sample and *F(R)* is the Kubelka–Munk function.

Electron excitation from the valance band to conduction band gives a measure of the optical band gap which can be estimated using the relation *(F(R) hυ)^n^ = A(hυ-E_g_),* where *n* = 2 and ½ for directly allowed and indirectly allowed transition respectively, *A* is the constant, and *hυ* is the photon energy [38]. Extrapolation of the linear part of the curve in Figure 6b to *(F(R)hυ)^2^ =* 0 leads to the direct band gap energy. The decrease in band gap can be attributed to the concentration of free carriers as well as impurity effect when NiO is doped. Normally, NiO and Li doped NiO are p-type semiconductors containing an excess of oxygen. On doping, some of the Ni^2+^ are replaced with Li^+^. This results in an increase in the concentration of Ni^3+^ and holes too. According to the Moss–Burstein effect [39], this should have caused a shift in the reflectance edge towards a higher photon energy. However, in the present case, the band gap decreases due to the following two Coulomb forces: (i) the exchange and correlation energy between the holes and the electrons in the valence and conduction band, respectively; and (ii) interaction between holes and impurity ions.

**Figure 7:**
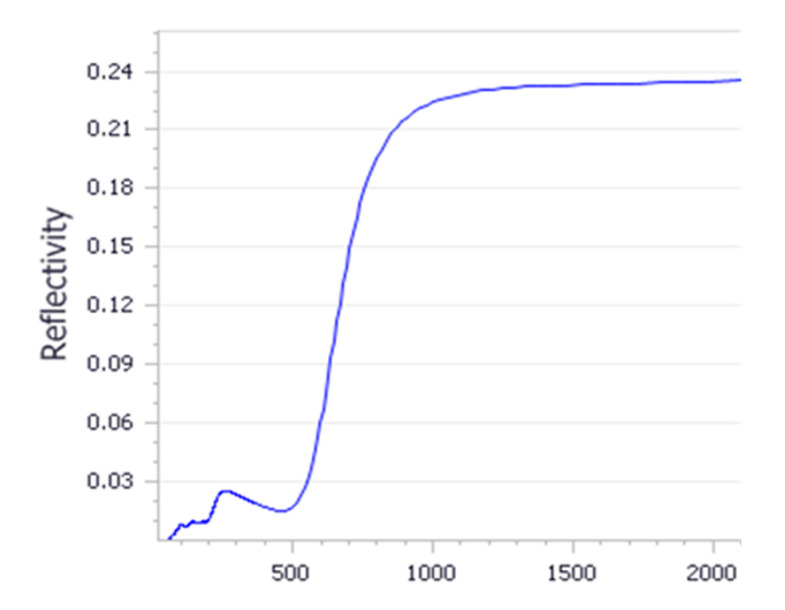
Simulated reflectivity as a function of wavelength.

Optical properties for NiO and Li doped systems were calculated using the first-principle density functional theory method. Simulated reflectivity as a function of wavelength is presented in Figure 7, along with experimental measurements. As observed experimentally, the nanoparticle depicts an absorption edge in the ultraviolet region. Optical absorption curve showed that the nanoparticles absorbance is attributed by peaks ranging from 100 to 400 nm. This suggests that the Li doped NiO particles can be stable at wavelengths below 400 nm. Furthermore, simulations showed a long wavelength activity in the visible light region.

### 3.5. Photoluminescence Studies (PL)

**Figure 8:**
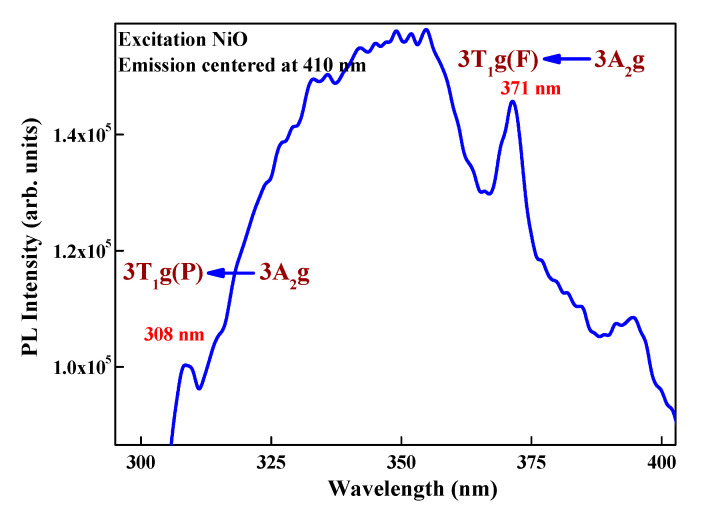
Excitation spectrum of NiO emission wavelength monitored at 410 nm.

The photoluminescence excitation spectra (Figure 8) of the prepared samples at emission wavelength of 410 nm exhibit a prominent broad band at 308 nm along with an intense peak at 371 nm, corresponding, respectively, to spin-allowed 3T_1_g(F) ← 3A_2_gand3T_1_g(P) ← 3A_2_g transitions of Ni^2+^ ions [40]. The intensity and position of these bands are characteristic of octahedral Ni^2+^.

**Figure 9:**
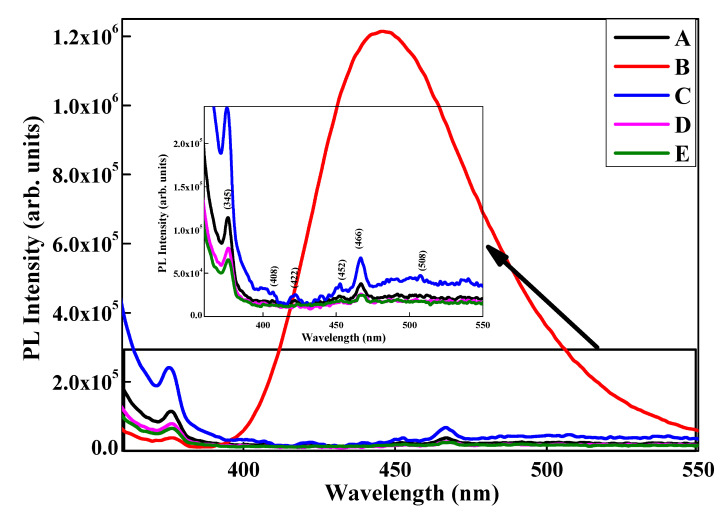
Emission spectra of different NiO samples excited at 308 nm.

The PL emission spectra (Figure 9) of the samples, upon excitation at 308 nm, shows two PL peaks that are obvious at 345 and 466 nm, corresponding to ultraviolet emission (340–400 nm) and blue emission (450–495 nm), respectively. Similar observations have been made in the literature citing PL studies on NiO nanoparticles [41,42].The origin of photoluminescence peaks can be attributed to electronic transitions involving 3d^8^ electrons of the Ni^2+^ ions [43].The presence of Li^+^ resulted in the formation of several shoulder peaks: at 402 and 422 nm (violet emission), 452 nm (blue emission) and 508 nm (green emission).The violet emission peaks are probably due to the transition of trapped electrons at interstitial Ni to the valence band. The blue emissions are due to the recombination of electrons from the Ni^2+^ vacancy to the holes in the valence band [41]. The cause of the green emission peak is not yet clear, as some authors cite it to be due to increases in Ni vacancies [44] whereas others relate it to the oxygen vacancies [45]. The addition of lithium influences the PL spectra profoundly; samples D and E have a lower emission intensity, whereas sample C has a higher emission intensity, than pristine NiO. The UV and visible emission peak intensity is dependent on radiative recombination. At a lower concentration of dopant, the radiative recombination process is higher, emitting more energy and thereby intensifying the peak. A small amount of dopant results in the replacement of Ni^2+^ with Li^+^, generating one hole in the valence band to maintain charge neutrality [46]. However, as the dopant concentration increases, it induces higher defects. This probably leads to non-radiative recombination, which subsequently reduces the peak intensity [47]. Interestingly, the sample B (NiO–surfactant) exhibits a high-intensity broad peak from 400–550 nm with maxima at 440 nm. A similar result has been reported by Wang and his group for NiO synthesized using dodecylamine as surfactant. The authors have attributed the broad nature of PL emission to the multilayer structure formed by layered NiO–surfactant superlattices. This kind of structure is expected to influence the chemical and physical properties of NiO to a large extent [48]. The oxygen vacancies may interact with interfacial capping surfactants, forming a series of metastable energy levels within the band gap. The long lifetime and dipole allowed for by transitions in these metastable energy levels induces the interfacial effect between NiO and the surfactant. Such unusual room temperature photoluminescences have also been previously observed by Zou and his group for nanoparticles coated with stearic acid [49] and by Bai and co-workers for mesolamellar TiO_2_ structures [50]. The intensity emission peak indicates enhanced photoluminescence intensity with high charge transfer resistance and, hence, a decrease in the electrochemical behavior further confirmed by CV and EIS analysis.

### 3.6. Cyclic VoltammetryAnalysis

**Figure 10:**
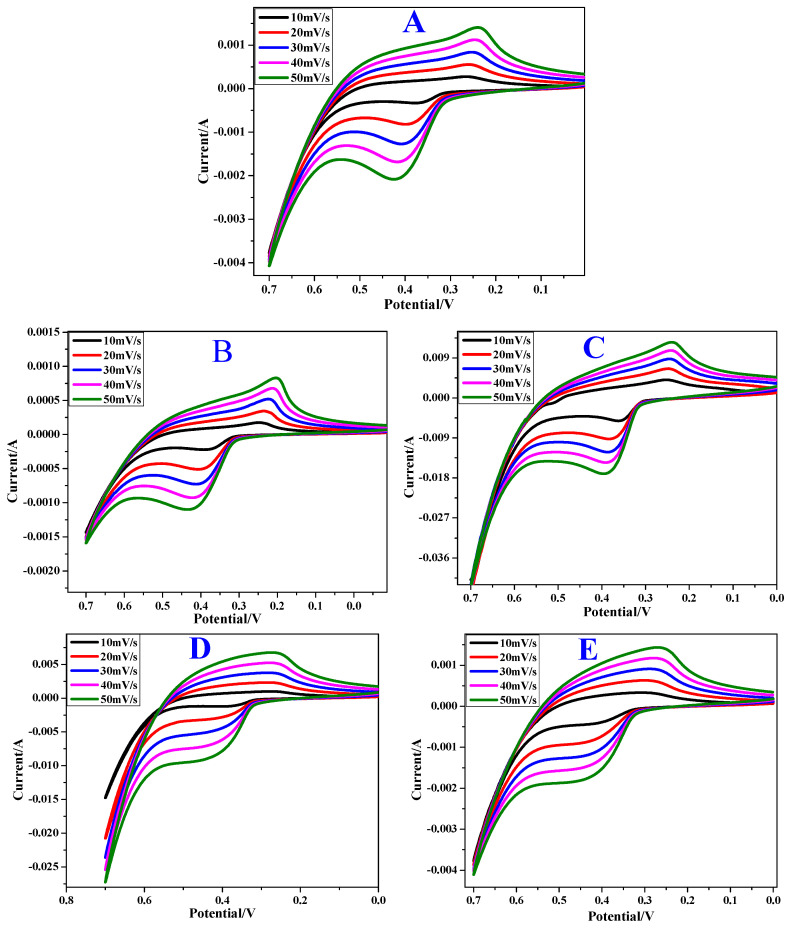
Cyclic voltammogram of (**A**) pristine NiO, (**B**) pristine NiO with PEG, (**C**) NiO with PEG and 1 wt% Li, (**D**) NiO with PEG and 2 wt% Li, (**E**) NiO with PEG and 5 wt% Li at various scan rates.

The cyclic voltammetric studies of the synthesized samples are shown in Figure 10. The CV curve has definite symmetric anodic and cathodic peaks, indicating the good reversibility of the redox reactions. However, by increasing the scan rates, no significant change was observed within the material. As evidenced by CV studies, the electrochemical process of NiO electrodes is limited by the proton diffusion through the lattice [51,52,53]. By taking into account the difference between the oxidation potential (*E_O_*) and the reduction potential (*E_R_*) at a scan rate of 10 mV/s, the reversibility of the electrode reaction was measured. The reversibility increases as the *E_O_* − *E_R_* value decreases. From Table 2, it is evident that the reversibility of the electrode reaction was maximum for NiO sample prepared with 1% Li dopant. The electrode reactions in NiO proceed according to the following reaction:

(6) $$\mathrm{NiO}\;\overset{\mathrm{Oxidation}}{\underset{\mathrm{Reduction}}{⇌}}{\;\mathrm{Ni}}^{2+}+{\;O}_{2}^{2-}$$

The anodic and cathodic peaks indicate the oxidation of Ni^0^ into Ni^2+^ and reduction of Ni^2+^ into Ni^0^, respectively. The quasi-reversible electron transfer process seen in the CV curve indicates the measured capacitance based on the redox mechanism [54]. The peak heights or the area of the CV curve for doped samples are larger than pristine samples, denoting a high amount of stored charge. This could be related to the smaller size of the doped samples resulting in a higher surface area. However, NiO with 2% Li exhibits a smaller CV curve when compared to the undoped samples. The reason for this deviation is unclear.

The quantity of current generated by electrode C is comparatively higher, and is least for electrode B. This suggests that 1% Li plays a crucial role during lattice formation which is manifested by generation of higher current when compared to the rest of the doped oxides. On the other hand, the presence of PEG as a surfactant during the synthesis has a great impact on the surface morphology. This is explicit by a higher PL intensity, as well as a decrease in the electrode reversibility. Probably, the surfactant enclosing NiO diminishes the development of the current.

### 3.7. Impedance Studies

**Figure 11:**
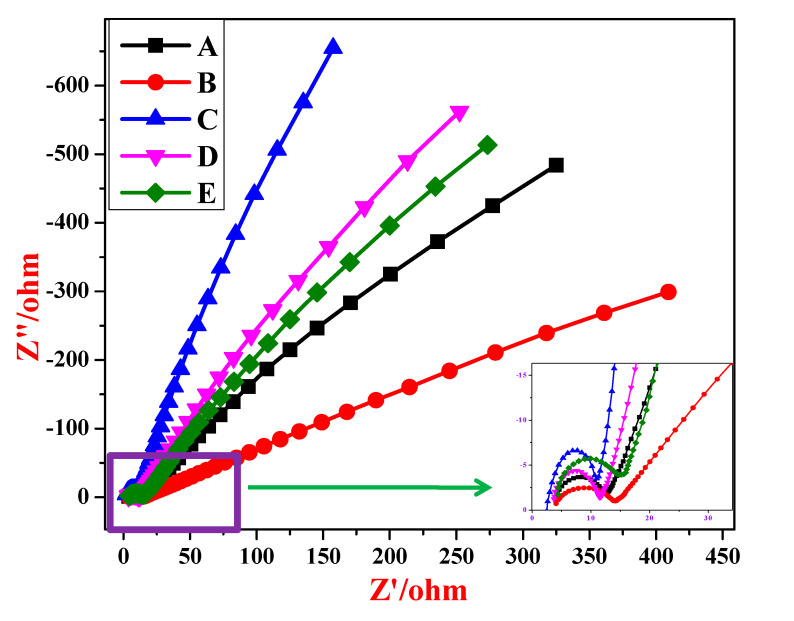
Nyquist plots of samples A, B, C, D and E.

The electrochemical impedance measurements were carried out in the frequency range of 1 Hz to 1 MHz at 5 mV steady state amplitude. The corresponding Nyquist plots are shown in Figure 11. The plots suggest a larger impedance for electrode A (semicircle with a bigger diameter) while the impedance of the electrode C was found to be smaller (Table 3), as is evident by the shifting of the imaginary line towards Y-axis. Consequently, electrode C manifests higher discharge rates and capacitance. The increase in the capacitance can also be attributed to the combined effect of the electric double-layer capacitance on the high surface area of AC and pseudocapacitance via the intercalation/extraction of Li ions in NiO lattice.

Since the Nyquist plots reveal the presence of depressed semicircles with a centre below the real axis at higher frequencies, it becomes necessary to use a constant phase element (*Q*_1_) to fit the data into an equivalent circuit. The impedance of *Q*_1_ can be described [55,56] as

(7) $$Z_{CPE}=\frac{1}{Y{\left(j\omega \right)}^{n}}$$

where *ω* is the angular frequency in rad s^−1^, *Y* and *n* are adjustable parameters of constant phase element (*Q*_1_). For double layer capacitance, the value of *n* = 1, for resistance and Warburg diffusion *n* = 0 and *n* = 0.5, respectively.

**Figure 12:**
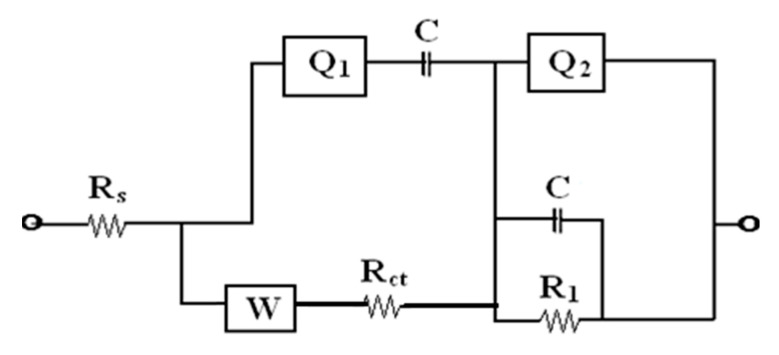
Equivalent circuit for Nyquist plot of samples A, B, C, D and E.

The equivalent circuit for the Nyquist plots of impedance measurements of NiO electrodes A–E is shown in Figure 12. In the given circuit, the high frequency region corresponds to solution resistance (*R_s_*) at the electrode–electrolyte interface. The semicircles are attributed to an interfacial charge transfer resistance (*R_ct_*) or polarization resistance (*R_p_*) to which the double-layer capacitance (*C*) is connected in parallel. The low frequency region straight line is represented by the Warburg element (W) in series with *R_ct_*. Warburg element is an estimation of the redox reactions occurring in the system, i.e., the diffusion of electrons from the working electrode and deposition of nickel ions from the electrolyte into the pores on the electrode surface [57], the electrolytic diffusion of ions takes place during the transition from the high-frequency semicircle to the mid-frequency point [58]. The constant phase element (*Q*_1_) lies parallel to the charge-transfer resistance (*R_ct_*), as does the low frequency capacitance (*Q*_2_) to the leakage resistance (*R_l_*).

EIS-fitted circuit parameters are tabulated in Table 3. The data were obtained by fitting the experimental data as per the equivalent circuit. A decrease in *R_ct_* and an increase in *C_dl_* indicates an enhancement in the electrochemical activity of the electrode. It is evident from Table 3 that the electrochemical activity of the electrode *C* (1% Li dopant) was higher, which may be attributed to the Li grains’ effectiveness in current collection and further improves the charge transfer process on interface between electrolyte and electrode. However, this effect subsides with increasing Li concentration. On the other hand, due to the surfactant effect there is an increase in the *R_ct_* value and a decrease in *C_dl_* value. This is consistent with the PL emission results.

## 4. Conclusions

In summary, undoped and Li doped NiO nanoparticles have been successfully prepared by microwave technique. The nanostructures were found to be crystalline in nature, with a cubic structure. The crystallite size decreased after the introduction of an Li dopant as revealed from X-ray diffraction studies. A decrease in band gap and an ultraviolet-blue emission along with small amount of green emission suggests that the photoluminescence of NiO nanomaterials can be tuned by doping. The optical results were further confirmed by computational modelling. From CV measurements, it was observed that the reversibility of NiO sample was maximum with 1% Li dopant. The same electrode also had enhanced electrochemical property with the charge transfer resistance reducing to 5.592 × 10^−8^ Ω. Through the present work, the optical and electrochemical behavior of Li doped NiO have been successfully demonstrated. We believe that these nanostructures can be applied to nanoscale electrochromic and electro-optical devices for various consumer and industrial applications.

## Figures and Tables

**Table 1:** Crystallite size calculated using X-ray diffractometer.

Sample	Size (nm)
A	3.08
B	2.47
C	2.36
D	2.43
E	2.80

**Table 2:** Oxidation potential (*E_O_*), reduction potential (*E_R_*) and the difference between *E_O_* and *E_R_* of different NiO electrodes.

Name of the Electrode	*E_O_*	*E_R_*	*E_O_* − *E_R_*
A	0.419	0.240	0.179
B	0.428	0.201	0.227
C	0.392	0.238	0.154
D	0.419	0.253	0.166
E	0.428	0.254	0.174

**Table 3:** The EIS fitted circuit parameters of *R_Ct_* and *C_dl_* values.

Name of the Electrode	*R_Ct_* (Ω)	*C_dl_* (F)
A	9.146 × 10^−3^	1.147 × 10^−4^
B	3.549 × 10^−2^	1.404 × 10^−5^
C	5.592 × 10^−8^	1.72 × 10^−3^
D	1.277 × 10^−4^	1.239 × 10^−5^
E	8.091 × 10^−4^	1.201 × 10^−7^
